# Characterization and Genomic Analysis of a Bacteriophage with Potential in Lysing *Vibrio alginolyticus*

**DOI:** 10.3390/v15010135

**Published:** 2022-12-31

**Authors:** Jingyun Fu, Ying Li, Lihong Zhao, Chunguang Wu, Zengguo He

**Affiliations:** 1 College of Medicine and Pharmacy, Ocean University of China, Qingdao 266003, China; 2 Laboratory of Microbial Engineering, Marine Biomedical Research Institute of Qingdao, Qingdao 266071, China; 3 Bioantai Biotechnology Co., Ltd. of Qingdao, Qingdao 266000, China

**Keywords:** *Vibrio alginolyticus*, vibriosis, phage therapy, *Vibrio* phage, Vibrio control

## Abstract

*Vibrio alginolyticus* is one of the major pathogens causing vibriosis to a variety of aquatic animals as well as bringing about severe food safety concerns. Nowadays, phage therapy has received increasing attention as an alternative to the antibiotics that have being limited for use in aquaculture industries. In this work, a potent bacteriophage, vB_ValM_PVA23 (PVA23), which efficiently infects pathogenic strains of *V. alginolyticus*, was isolated from sewage water and characterized by microbiological and genomic analyses. Based on the transmission electronic observation, the phage was characterized to be the *Myoviridae* family. It has a latent period of 10 min and a burst size of 203 PFUs/infected bacterium, and was stable over a broad pH range (5.0–11.0) and a wide temperature span (−80 °C to 60 °C), respectively. Genome sequencing results show that PVA23 has a 246,962-bp double-stranded DNA with a G + C content of 41.25%. The lab and plant shrimp farming trials demonstrated that phage preparation derived from PVA23 out-performed the chemical disinfectant iodine treatment in the prevention of *V. alginolyticus* propagation, and the phage application could rapidly yet significantly reduce the level of *V. alginolyticus* in the pond within 12 h, with negligible rebound observed. These results suggests that phage PVA23 has the potential to be used as an anti-*V. alginolyticus* agent in aquaculture industries.

## 1. Introduction

Aquatic products are one of the major sources of food protein for humans in the world. With the rapid thriving of aquatic farming worldwide, the diseases derived from fatal aquatic bacterial pathogens, such as *Vibrio*, are posed as a huge threat. Some strains of *Vibrio* produce multiple virulence factors, such as hemolysin, caseinase, lipase, gelatinase and phospholipase, which are detrimental to aquatic animal health [1,2]. Among them, *V. alginolyticus* is a major pathogenic species that is widely distributed in coastal waters worldwide. It is well known that *V. alginolyticus* causes vibriosis to aquatic animals like shrimp, fish and shellfish, leading to mass mortality of economic marine animals as well as severe economic losses [3,4]. *V. alginolyticus* also brings about food safety concerns by polluting the sea food chains and producing toxins that are dangerous to human beings [5,6].

Currently, application of chemical agents, such as chemical disinfectants and antibiotics, is still the routine approach to control *Vibrio* in aquatic farming practice. Except for disease prevention, chemical disinfectants are also used widely for clearing the pond water, bathing the seedling, disinfecting pond mud and so on. However, it was found that the chemical disinfectants could adversely affect the growth of the farming animals as well as the beneficial microbes due to their toxic nature and non-selective killing effects [7]. Furthermore, the antimicrobial activities of chemical disinfectants are concentration dependent, so the routine water change over and the exposure to continuous airing may lower their concentration and largely discount their activity. On the other hand, the long-term overuse of the chemical antibiotics has led to adverse consequences such as the accumulation of chemical residuals, the emergence of multidrug resistances of *Vibrio*, and the microbial changeover, which not only arouses food safety issues, but also makes aquatic farming unsustainable [8]. Therefore, demand of safer alternatives for *Vibrio* control is on the rise.

Bacteriophages are viruses that can infect and kill their specific host bacteria and regulate bacterial populations by inducing bacterial lysis [9]. The use of phage therapy can be traced back to a century ago, and later on it was widely accepted, thanks to a panel of advantages over traditional antibiotic therapies [9]. Phage therapy has been considered as a potential alternative to antibiotic therapy in treating bacterial infections, and it had been successfully used to control several pathogenic infections in humans, and veterinary and agricultural settings [10]. Recently, some investigators have explored phage therapy for aquatic farming application as well as for bio-preservation use in food industries [8,11,12]. A panel of investigations have confirmed that preparations of phage or phage cocktails could effectively kill a variety of pathogenic *Vibrio* isolated from the ponds that farm different aquatic animals, such as shrimp, fish, oyster and sea cucumber [13,14,15,16,17]. Phage therapy has also been confirmed to be active against some dangerous emerging pathogens like multidrug-resistant bacteria in fish farming [8]. Furthermore, phage therapy has also been documented for the inhibition of biofilms formulated by *Vibrio* [18]. In short, phage therapy is becoming a promising antibiotic alternative for use in aquaculture.

In this study, a phage PVA23 was isolated from local sewage samples for its high efficiency in lysing potential against *V. alginolyticus*. The phage was characterized genomically and phenotypically, and its application against *V. alginolyticus* was evaluated in pond trials in parallel with disinfectants commonly used in farming practice.

## 2. Materials and Methods

### 2.1. Isolation and Identification of V. alginolyticus Strains

*V. alginolyticus* strains used in this study were isolated from both diseased fishes and shrimps; the wastewater samples were collected from aquaculture ponds in different regions of China using the methods as reported [19]. Fish and shrimp samples were initially disinfected with 0.1% (w/v) benzalkonium chloride and then rinsed with sterile 0.85% (w/v) saline. Under aseptic conditions, animal tissues were ground, homogenized in a glass homogenizer with 5 mL of sterile saline, then the juice was pipetted promptly onto thiosulphate citrate bile salts sucrose agar culture medium (TCBS, Hope Biotechnology Co., Ltd. of Qingdao, Qingdao, China) plates. The plates were incubated at 37 °C for 24 h. Morphologically uniform yellow colonies with a diameter of about 2–4 mm were picked and thread onto new TCBSs to obtain pure cultures. The species of these purified strains were identified by a 16S rRNA PCR using the following universal primers: 27F: 5′-AGAGTTTGATCCTGGCTCAG-3′ and 1492R: 5′-GGCTACCTTGTTACGACTT-3′. The PCR products obtained were sequenced and compared with the reference 16S rRNA sequence of *V. alginolyticus* in the GenBank. All identified isolates were incubated at 37 °C with shaking at 170 rpm in 2216E liquid medium (Hope Biotechnology Co., Ltd. of Qingdao, Qingdao, China) and stored at −80 °C with 20% glycerol.

### 2.2. Detection of Virulence Genes of the V. alginolyticus Isolates

A panel of common virulence genes of *V. alginolyticus*, including collagenase gene (*Col*), alkaline serine protease gene (*AspA*), helmolysin genes (*tlh*, *tdh* and *trh*), flagellar filament protein gene (*flaA*), outer membrane protein genes (*ompW* and *ompK*), ferric uptake system gene (*fur*), gyrase B subunit (*gryB*) and virulence-regulating genes (*toxR* and *toxS*), were detected by PCR assay [2,20] (Table 1). The PCR amplification was performed under the following conditions: initial denaturation at 94 °C for 4 min, followed by 35 cycles of 94 °C for 1 min, 60 °C for 1 min, and 72 °C for 1.5 min; and a final extension at 72 °C for 8 min. Amplified PCR products were visualized on a 1% agarose gel stained with ethidium bromide, running at 90 V for 25 min. The presence of virulence genes was confirmed by gel electrophoresis showing the corresponding target band by the gel imaging system after repeating the PCR three times.

### 2.3. Isolation, Purification and Propagation of Bacteriophages

Isolation of phages was conducted using the conventional double-layer agar method [13]. The *V. alginolyticus* that isolated with the most virulent genes from the above experiment were then used as hosts to isolate their specific phages. The sewage sample was collected from a large-scale aquatic market in Qingdao, China. First, sewage water (20 mL) was centrifuged at 8000× *g* for 10 min, and filtered through a 0.22 µm membrane to get rid of bacteria. Next, 5 mL of the filtrate and 5 mL of *V. alginolyticus* culture (10^7^ CFU/mL) were added to 50 mL of sterilized 2216E medium. The mixture was incubated at 37 °C with orbital shaking for 6 h to enrich possible phages, then it was centrifuged at 8000× *g* for 10 min and filtered through a 0.22 µm membrane to remove the remaining bacteria. 100 µL of the filtrate, mixed with 100 μL of *V. alginolyticus* culture, was poured into 5 mL of molten soft 2216E upper agar (2216E liquid medium with 0.7% agar) and then poured onto the surface of a prepared and cooled 2216E lower plate (2216E liquid medium with 1.5% agar). The phage plaque’s appearance was observed after the overnight incubation at 37 °C.

A single plaque was picked into 1 mL of sterilized PBS buffer (8 g NaCl, 0.2 g KCl, 1.44 g Na_2_HPO_4_·12H_2_O, 0.24 g KH_2_PO_4_, 0.11 g CaCl_2_, adding water to 1000 mL and adjusting pH to 7.2 with Na_2_HPO_4_·12H_2_O or KH_2_PO_4_) and bathing in water at 40 °C for 30 min to harvest phages. Then, the phage-containing PBS buffer was centrifuged at 12,000× *g* for 5 min and filtered through a 0.22 µm membrane. This filtrate was diluted at a 10-fold gradient with PBS buffer to a suitable multiple which could obtain single plaques on the double-agar plate. The single-plaque isolation process by the double-layer agar method was repeated four more times until plaques of uniform size, shape and clarity were obtained.

The lysate of the pure single plaque in 100 µL PBS was mixed with an equal amount of fresh culture of the host strain, and then was added into 5 mL of 2216E tube and incubated at 37 °C with shaking at 170 rpm to get a phage proliferation solution. Cell debris of the solution was removed by centrifugation and filtration. The bacteriophage titer of the filtrate was determined using the double-layer agar method and expressed as plaque forming units (PFU) per mL filtrate. The proliferation solution was stored at 4 °C for further experiments.

### 2.4. Screening of Lytic Bacteriophages

The screening of lytic bacteriophages was based on the lytic capability and the host range against the *Vibrio* strains. Here, the host ranges of the isolated phages were determined by the method of standard spot tests assay [22]. All *V. alginolyticus* isolates from above experiment and the reference strain *V. alginolyticus* ATCC 17749 (purchased from Guangdong Microbial Culture Collection Center) were tested. Briefly, 100 µL of each bacterium (10^7^ CFU/mL) was added to 5 mL of molten soft 2216E upper agar and then poured onto the surface of a prepared and cooled 2216E lower plate. 2 µL of PVA23 stock (10^9^ PFU/mL) was spotted onto the lawn and incubated at 37 °C for 12 h. The bacterial hosts of a tested phage were recorded if a lysis circle could be observed in the plate. The experiments were conducted in triplicate.

### 2.5. Determination of Host Ranges of Phage PVA23

The phage PVA23 was selected for further experiments. The host range of PVA23 was determined by two methods of standard spot tests assay and efficiency of plating (EOP) assay [23]. All *V. alginolyticus* isolates from above experiment and the reference strain *V. alginolyticus* ATCC 17749 (purchased from Guangdong Microbial Culture Collection Center) were tested. The spot tests of PVA23 have been done in the above section of screening of lytic bacteriophage. For the EOP assay, phage host ranges were identified by the EOP, which was measured by comparing titers between the test strains and *V. alginolyticus* VA15 as a reference. The EOP of bacteriophage is generally close to 1, with EOP ≥ 0.5 meaning high production, 0.1 ≤ EOP < 0.5 meaning medium production, 0.001 ≤ EOP < 0.1 belonging to the low production, and EOP ≤ 0.001 meaning no production [24]. The experiment was conducted in triplicate.

In addition, other species of *Vibrio* and non-*Vibrio* commonly found in pond water and saved in our laboratory, were also tested to further determine the host range of phage PVA23 using the method of standard spot tests assay. The experiment was conducted in triplicate.

### 2.6. Transmission Electron Microscopy of Phage PVA23

The morphology of PVA23 was characterized by using transmission electron microscopy [13]. Phage particles were precipitated with 10% polyethylene glycol 8000 (PEG 8000) at 4 °C overnight, centrifuged at 10,000× *g* for 15 min, and subsequently suspended in PBS buffer. One drop of the concentrated phage particles was spotted onto the surface of carbon grids for 15 min and then negatively stained with 2% phosphotungstic acid for 10 min. The phage was then observed using a Hitachi H-7700 biological transmission electron microscope (Hitachi High-Technologies Corporation, Tokyo, Japan) at an accelerating voltage of 80 kV. It was classified and identified on the basis of International Committee on Taxonomy of Viruses (ICTV) guidelines.

### 2.7. Determination of Optimal Multiplicity of Infection (MOI) and One-Step Growth Curve of Phage PVA23

In order to determine the optimal MOI of PVA23, 100 µL of serial dilutions of stocked solution (10^9^ PFU/mL) and 100 µL of the host *V. alginolyticus* VA15 culture in logarithmic phase (10^7^ CFU/mL) were added to 5 mL of 2216E broth to generate the MOI at 0.000001, 0.00001, 0.0001, 0.001, 0.01, 0.1, 1, 10 and 100, respectively. The mix was incubated at 37 °C with orbital shaking for 12 h. Then, the titers of the phage at different MOIs were determined by the double-layer plate method. The one with the highest titer was the optimal MOI. The experiment was conducted in triplicate.

In this work, the one-step growth analysis of PVA23 was performed by previous descriptions [25] with some modifications. First, PVA23 stock (10^9^ PFU/mL) was added to the host *V. alginolyticus* VA15 culture in logarithmic phase (10^7^ CFU/mL) at a MOI of 1. After incubation at 37°C for 10 min, the broth was centrifuged at 12,000× *g* for 30 s to remove unabsorbed free phage. Next, the pellets were washed with 2216E for twice at 37 °C, and the suspension was transferred to 20 mL of 2216E followed by immediate incubation at 37 °C with shaking. This moment was defined as t = 0 s, and 200-μL sample was collected every 10 min up to 180 min. The titer of phage particles was conducted using the double-layer agar method. All experiments were performed in triplicate. The burst size of PVA23 was calculated as the ratio of the final count of liberated phage particles to the initial count of infected bacterial cells.

### 2.8. Determination of Thermal and pH Stability of Phage PVA23

To determine the thermostability of PVA23, stocked solutions (10^9^ PFU/mL) were treated at different temperatures of 40 °C, 50 °C, 60 °C, 70 °C, 75 °C and 80 °C, respectively. Aliquots of 200-μL were taken at 20, 40, and 60 min during the incubation at each temperature, respectively. Then, the double-layer agar method was used to calculate phage titer against *V. alginolyticus* VA15. The experiment was conducted in triplicate.

To evaluate pH stability of PVA23, aliquots of 1 mL stocked solution (10^9^ PFU/mL) was treated at 37 °C for 2 h with 10 mL PBS buffers with varied pH values (2–13). Then the double-layer agar method was used to calculate the phage titer. The experiment was conducted in triplicate.

### 2.9. Determination of Chloroform and Ultraviolet (UV) Sensitivity of Phage PVA23

To determine the chloroform sensitivity of the phage, 5 mL of PVA23 stock (10^9^ PFU/mL) was mixed with chloroform to get final concentration at 1%, 2%, 3%, 4% and 5%, respectively, and incubated at 37 °C. The mix was incubated at 37 °C for 30 min by shaking. Then, 200-μL aliquots was taken for phage titer measuring. The phage stock solution without chloroform treatment was used as the control. The experiment was conducted in triplicate.

To determine the impact of Ultraviolet (UV) irradiation, 20 mL of PVA23 stock (10^9^ PFU/mL) was poured into individual 8 cm sterile glass plate and was allowed to be exposed to UV irradiation (20 W, 30 cm) for varied times, respectively. Aliquots (100 µL) of each was withdrawn every 3 min within 30 min. A phage particle not subjected to UV irradiation was used as the control. The experiment was conducted in triplicate. Phage titer was measured to check the ultraviolet (UV) sensitivity of PVA23.

### 2.10. Assay of Lysis Activity In Vitro of Phage PVA23

The *V. alginolyticus* VA15 culture (in logarithmic phase,10^7^ CFU/mL) was mixed with a 10-fold serial dilution of the phage stock (10^9^ PFU/mL) to make MOIs at 0.01, 0.1, 1 and 10, respectively. The mix was incubated at 37 °C for 300 min with orbital shaking. Host culture untouched with the phage was served as the control. Aliquots were collected at 10-min intervals and the optical density (OD_600_) was measured by a UV-vis spectrophotometer. The experiment was conducted in triplicate.

### 2.11. Assay of Bacterial Phage-Insensitive Mutation Frequency (BIMF) of Phage PVA23

To determine bacterial phage-insensitive mutation frequency (BIMF), PVA23 was used to lyse the host *V. alginolyticus* VA15 for resistance observation by following the method previously described [26,27]. The fresh cultures of VA15 in logarithmic phase (10^7^ CFU/mL, 100 µL) was used to co-culture with PVA23 stock (10^9^ PFU/mL, 100 µL) at 37 °C for 10 min, the host bacterium without phage was used as the control. After serial dilution, the mixture was coated on TCBS plates and incubated at 37 °C for 18 h, and the number of phage-resistant colonies in the plate was recorded (the number of assumed insensitive colonies). Then, all the resistant colonies were picked for 2216E broth, and tolerance to PVA23 was confirmed by the spot assay. The number of colonies without lysis circle was recorded (the number of determined insensitive colonies). BIMF is the ratio of the number of determined insensitive colonies to the total number of colonies. The experiment was conducted in triplicate.

### 2.12. DNA Extraction, Genome Sequencing and Assembly of Phage PVA23

First, the concentrated phage PVA23 particles were treated with DNase I and RNase A (New England BioLab, England) followed by incubating at 37 °C for 30 min to remove any exogenous host genomic DNA and RNA. Then, phage PVA23 genomic DNA was extracted using the λ Bacteriophage Genomic DNA Rapid Extracted Kit (Yuanye Biotechnology Co., Ltd. of Shanghai, Shanghai, China) following the manufacturer’s instructions. The purified phage PVA23 DNA was performed on an Illumina NovaSeq sequencing platform (Weilai Biotechnology Co., Ltd. of Qingdao, Qingdao, China). The filtered reads were assembled using SOAPdenovo by the default parameters. The complete genome of phage PVA23 was finished and then manually inspected.

### 2.13. Genome Analysis and Phylogenetic Analysis of Phage PVA23

Prediction of all open reading frames (ORFs) of the genome sequence was performed using GeneMarkS [28]. Annotation and functional analysis were conducted using BLASTP algorithm against the non-redundant (nr) protein database of the National Center for Biotechnology Information (NCBI) [29]. The genome circle map was drawn by CGView server. The genome sequences of [20] other closely related homologous phages were downloaded from NCBI database, and similarities between the genomic sequences were determined using the average nucleotide identity MUMmer (ANIm) [30]. The putative tRNA-encoding genes were searched using tRNAscan-SE [31]. The genes for virulence and antibiotic resistance were detected by the VFDB database and the ARDB database [32], respectively.

To determine the taxonomy of PVA23, the phylogenetic trees based on the nucleotide sequences of the major capsid protein and the large terminase subunit were constructed using the ClustalW program in MEGA 7.0 software with the neighbor-joining method and 1000 bootstrap replications [33]. Comparative genomic analysis of PVA23 with the highest similar reference phages was performed using Easyfig 2.2 [34].

### 2.14. Application Evaluation of Phage PVA23 for Vibrio Control in Laboratory Shrimp Culture Trials

To validate the performance of the phage in controlling *Vibrio* in practical applications, 500 healthy 60-day-old shrimps (*Lenaeus vannamei*; weight = 10 ± 0.5 g), cultured in 20 L glass tanks under appropriate conditions with aquatic water temperature maintaining at 25 ± 1 °C during the study, were equally divided into five groups. Each group contained 100 shrimp and was then divided equally into two parallel subgroups as replicates (50 shrimp per subgroup). The water used in the glass tanks of all tested groups was seawater, and it was sterilized before the experiment. The *V. alginolyticus* VA15 was used to challenge shrimp in the experiment. To prepare the cells of *V. alginolyticus* VA15, the culture in logarithmic phase was spined down and washed with sterile saline three times and then resuspended to make a stock at a concentration of 2 × 10^9^ CFU/mL by measuring the optical density (OD_600_) and coating TCBS plates. A certain volume of VA15 suspension was added to each test group to reach the final concentration to 2 × 10^6^ CFU/mL in the water. After 1 h, group 2 was added with an iodic disinfectant (found in market) at dose of 5 mg/L, and group 3, group 4 and group 5 receiving varied amounts of PVA23 stock (2 × 10^10^ CFU/mL) to obtain MOI at 0.1, 0.01 and 0.001, respectively. Group 1 was the blank control and received no treatment. The water samples of test groups were taken every 2 h and the *Vibrio* content was measured by coating TCBS plates, and the experiment was conducted in triplicate. The cumulative mortality of shrimps was recorded daily during the experiment.

### 2.15. Application Evaluation of Phage PVA23 for Vibrio Control in Shrimp Farming Plant

The experiment was made in a shrimp farming plant located in Rizhao, China, which was farming for shrimps (*P. vannamei*, average weight = 8 g ± 0.6g), kept in 30-m^3^ pond under appropriate conditions (water temperature 26 °C ± 1 °C, salinity 25‰). The pond water in this plant was changed by 20% every day. To control the propagation of pathogenic *Vibrio*, the pond was regularly added with agents, such as povidone-iodine, complex iodine, chlorine dioxide and probiotic products, etc., which were stopped at least 72 h before the phage tests.

The experiments contained four groups, and the *Vibrio* contents of the ponds in each group were detected by coating TCBS plates before the start. Group 1 was the blank control receiving no control agent. Group 2 was supplemented with an iodic disinfectant (found in market) at a dose of 5 mg/L. Group 3 and group 4 were supplemented with PVA23 stock with MOI at 0.01 and 0.001, respectively. Each group of the tests repeated twice, with pond water sampled every 4 h and the *Vibrio* content checked.

### 2.16. Nucleotide Sequence Accession Number and Phage Preservation

Phage PVA23 was preserved in the China General Microbiological Culture Collection Center (Beijing, China) with CGMCC number of No.24119. The complete genome sequence of PVA8 have been submitted to the NCBI GenBank database under accession number of ON190025.

### 2.17. Statistical Analyses

Data were analyzed by one-way analysis of variance (ANOVA) using Graph Pad Prism 8.0. Significant differences were determined using Tukey's Multiple Comparison Test. Statistical significance was considered at the *p* < 0.05 level, and the results were expressed as mean ± standard deviation (SD).

## 3. Results

### 3.1. Profile of Virulent Genes of V. alginolyticus Isolates

**Figure 1:**
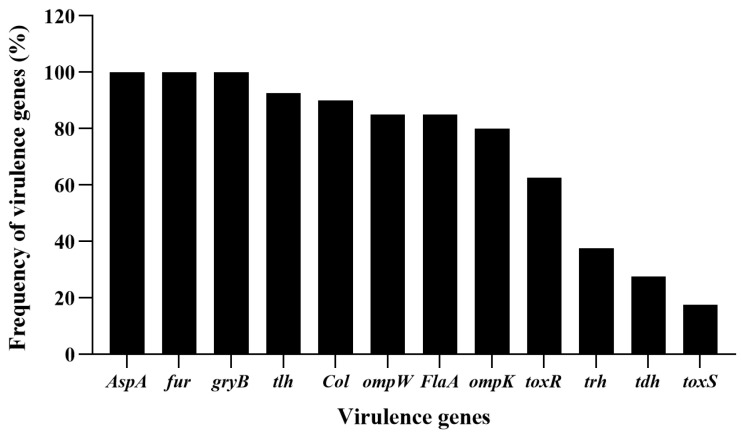
Frequency of virulent genes detected in the isolates of *V. alginolyticus*. Twelve virulent genes, *Col*, *AspA*, *tlh*, *tdh*, *trh*, *FlaA*, *ompK*, *ompW*, *fur*, *gyrB*, *toxR* and *toxS* were selected for PCR detection for the *V. alginolyticus* isolates.

In this work, in total, 40 *V. alginolyticus* strains were successfully isolated and identified. In order to investigate the pathogenic features of the isolates of *V. alginolyticus*, virulence-associated genes, collagenase gene (*Col*), alkaline serine protease gene (*AspA*), helmolysin genes (*tlh*, *tdh* and *trh*), flagellar filament protein gene (*flaA*), outer membrane protein genes (*ompK* and *ompW*), ferric uptake system gene (*fur*), gyrase B subunit (*gyrB*) and virulence-regulating genes (*toxR* and *toxS*) were checked by PCR assay. As shown in Figure 1, *AspA*, *fur* and *gyrB* were the most prevalent genes with the detection frequencies of 100% (40/40), followed by *tlh* (92.5%, 37/40), *Col* (90%, 36/40), *ompW* and *FlaA* (85%, 34/40), *ompK* (80%, 32/40), *toxR* (62.5%, 25/40), *trh* (37.5%, 15/40), *tdh* (27.5%, 11/40), and *toxS* (17.5%, 7/40), respectively. All the *V. alginolyticus* isolates caried virulent genes, and the majority carried multi-virulent genes. Among them, two isolates, *V. alginolyticus* VA15 and VA17, carried all the twelve virulent genes, thus they were selected as the presentative strains for later phage lysis testing as well as hosts for the phage screening.

The virulent genes used in the work are not all co-existing in the genome of *V. alginolyticus*, and their expression levels will change due to gene mutations or changes in the surrounding environment [2,20,35]. Therefore, the absence of some virulent genes in the above result did not imply the absence of the related genes in the genome, but the result was helpful in finding genetically stable *V. alginolyticus* strains showing more virulent genes [20].

### 3.2. Isolation and Screening of Lytic Bacteriophages using V. alginolyticus VA15 and VA17 as Hosts

Using *V. alginolyticus* VA15 and VA17 as hosts, six bacteriophages were successfully isolated and purified from the collected water samples, which were named *Vibrio* phage PVA21, PVA22, PVA23, PVA24, PVA25 and PVA26, respectively. These phages could form clear plaques on the double-layer plates with their host strains after overnight incubation at 37 °C, indicating that they were likely to be lytic phages against the hosts.

**Figure 2:**
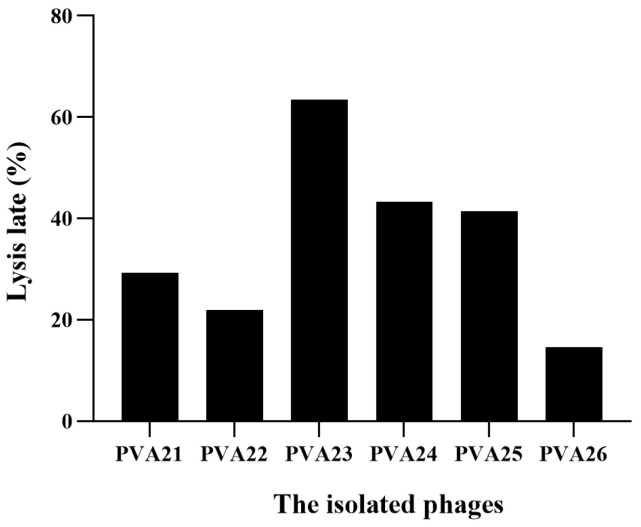
Lysis rate of the six isolated phages using the 41 *V. alginolyticus* strains.

The result of the host ranges of the six phages using the 40 *V. alginolyticus* isolates and the reference strain *V. alginolyticus* ATCC 17749 is shown in Figure 2 and Table 2. From the results, PVA23 had the broadest host range with the lysis rate of 63.41% (26/41), followed by PVA24 (19/41, 46.34%), PVA25 (17/41, 41.46%), PVA21 (12/41, 29.27), PVA22 (9/41, 21.95%) and PVA26 (6/41, 14.63%), respectively, and it also showed strong lytic ability to most of its hosts reflected by the maximum number of “+++” (Table 2). Therefore, phage PVA23 with *V. alginolyticus* VA15 as the host was selected for further experiments.

### 3.3. Host Ranges of Phage PVA23

To determine the inhibition activity of PVA23 more accurately, isolates of *V. alginolyticus* were further challenged by PVA23 to observe clear phage plaque using the EOP assay. According to the results of the spot inoculation test, about 63.41% (26/41) of the *V. alginolyticus* isolates could be inhibited by PVA23; however, the rate dropped to 56.1% (23/41) when EOP assay applied (Table 3). This difference could be because the EOP assay was more accurate in determining the host range of PVA23 as it excluded the abortive infections that may escape from the spot inoculation test. This result agreed with the recommendation on the use of progeny phages in the determination of host range for a given phage [24].

Furthermore, it was confirmed that isolate 15 was sensitive to PVA23 with relatively decent EOP of 1 (Table 3). Taking into account the 12 virulence genes carried, isolate 15 was used as the host strain of PVA23 hereafter.

In addition, the result of the host ranges of PVA23 using other species of *Vibrio* and non-*Vibrio* is shown in Table 4. Except *V. alginolyticus*, PVA23 could also lyse at least other two *Vibrio* species, and one of them was *Vibrio parahaemolyticus*. As expected, it did not exhibit any infectivity to bacterial species in genus other than *Vibrio*. This cross-species lytic ability and specificity for *Vibrio* species of phage PVA23 can be used to control different pathogenic *Vibrio* species and is a suitable choice for further study.

### 3.4. Morphology and Identification of Phage PVA23

**Figure 3:**
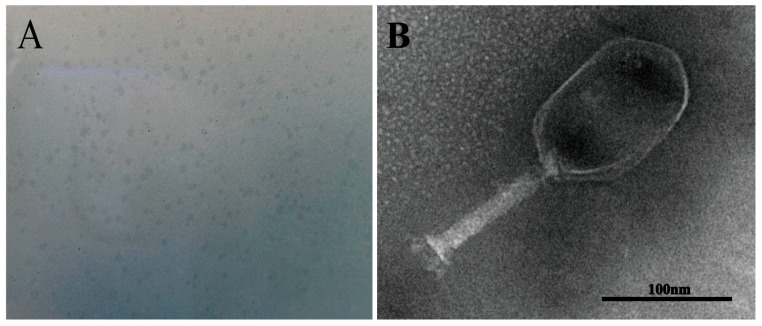
Phage plaques and transmission electron microscopy of phage PVA23. (**A**) Phage plagues of PVA23 formed on double-layer agar plate; (**B**) Transmission electron microscopy of PVA23, scale bar = 100 nm.

Phage PVA23 formed clear plaques on the lawn of the *V. alginolyticus* VA15, with an average diameter of about 0.5 mm after 8 h incubation (Figure 3A). The transmission electron microscopy image revealed that PVA23 had an elongated head (prolate capsid) about 134.57 nm long and 89.08 nm wide and a contractile tail about 115.14 nm long (Figure 3B). Therefore, PVA23 was identified as one member of the *Myoviridae* family, according to the transmission-electron-microscopy based on classification standards used in the International Committee on Taxonomy of Viruses (ICTV).

### 3.5. Biological Characteristics of Phage PVA23

**Figure 4:**
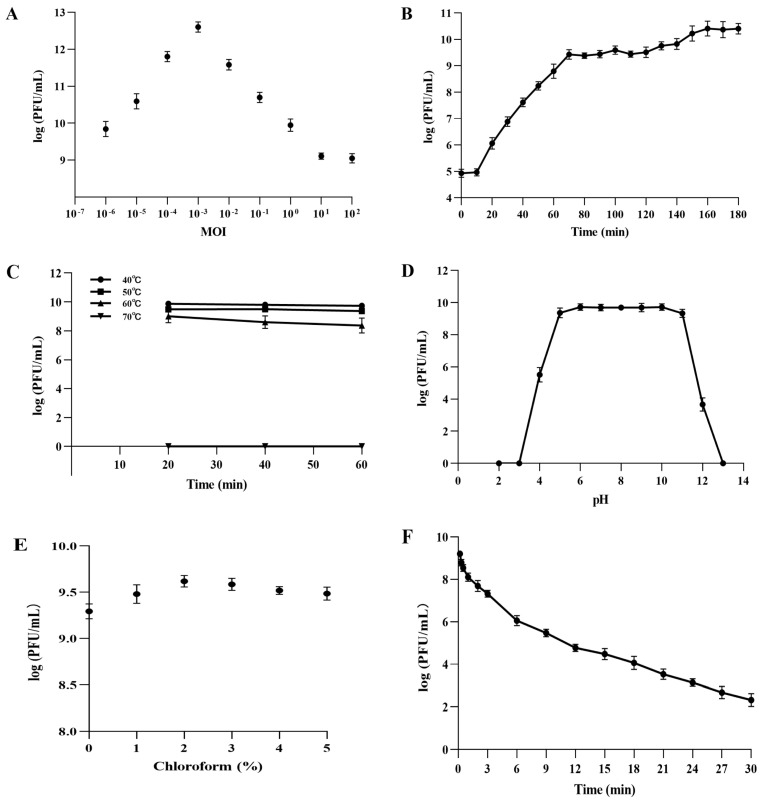
Biological characteristics of PVA23. (**A**) The MOI test of PVA23; (**B**) The one-step growth curve of PVA23; (**C**) The thermal stability test of PVA23; (**D**) The pH stability test of PVA23; (**E**) Chloroform sensitivity test of PVA23, and (**F**) Ultraviolet (UV) sensitivity test of PVA23.

The highest titer (4.26 × 10^12^ PFU/mL) of PVA23 was achieved at MOI of 0.001 (Figure 4A). Lowest MOI of PVA23 observed was as low as 0.000001, indicating that the phage had strong proliferative capacity.

PVA23 was confirmed to have relatively strong lysis ability by the one-step growth curve determination, in which the short latent time (10 min) and the large burst size (203 PFUs/infected cell) were demonstrated, respectively (Figure 4B).

The thermal stability assay showed that PVA23 was fairly stable at temperatures below 60 °C, but completely inactivated in temperatures at 70 °C (Figure 4C).

The pH stability test showed that PVA23 was stable over a broad pH range, from 5.0 to 11.0, for at least 2 h, but it was completely inactivated under extreme pH conditions (below pH 3.0 or above pH 13.0) (Figure 4D).

As shown in Figure 4E, PVA23 was not sensitive to the chloroform treatment, which indicated the phage capsid protein contained very limited or no lipids.

The survival curve of PVA23 under UV irradiation was depicted in Figure 4F. The results demonstrated that PVA23 was sensitive to the UV irradiation, by following a time based PFU declination trend as observed.

### 3.6. Lysis Activity in vitro of Phage PVA23

**Figure 5:**
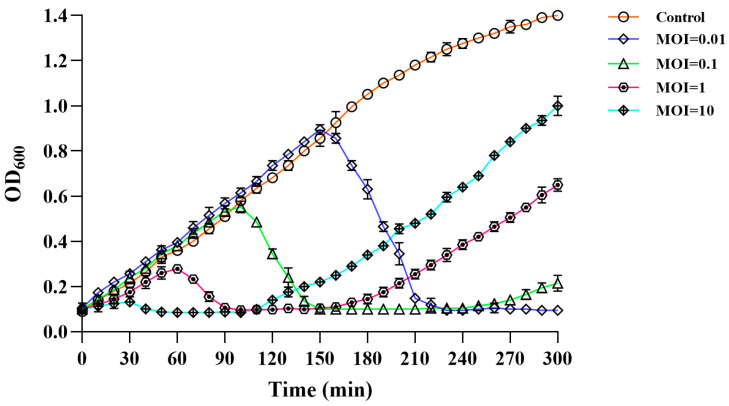
Lysis activity in vitro of phage PVA23 against the host organism VA15 at four different MOIs (10, 1, 0.1 and 0.01).

To evaluate the lysis potential of PVA23, in vitro tests were conducted using strain VA15 as the host with four MOIs at 0.01, 0.1, 1 and 10 applied, respectively (Figure 5). It was observed that the OD_600_ kept increasing and amounted to 1.399 within 300 min in the control, whereas the OD_600_ of the treatments reduced significantly after the initial growth in the first few hours, indicating the lysis of the cells was caused by PVA23. It was also noted that the lysis time was negatively correlated to the MOIs, with a rough S-shape OD_600_ trend observed for each MOI, respectively. The OD_600_ rebounding might reflect the establishment of host resistance to the phage infection via some undefined escaping ways, such as mutation or alteration. It was of interest that the higher MOI lead to faster rebounding in OD_600_ of the host.

### 3.7. Bacterial Phage-Insensitive Mutation Frequency (BIMF) of V. alginolyticus VA15 to Phage PVA23

To investigate the rebounding of the host bacteria, the BIMF test was conducted. As shown in Table 5, the BIMF of *V. alginolyticus* VA15 to PVA23 was about (8.48 ± 0.63) × 10^−7^, similar to those seen in other references [27]. Above results reasoned why the rebounding of the host bacteria in the phage application experiment were as observed, which implied that a certain mutation might occur when the target *Vibrio* strain was challenged with the phage. Thus, for the control of *V. alginolyticus* VA15, phage cocktails were suggested in practice to reduce the possible resistance derived due to mutations in the targeted *Vibrio*.

### 3.8. Genome Sequencing, Characterization and Analysis of Phage PVA23

The complete genome of PVA23 was 246, 962 bp long, and 99.49% of the reads was matched to the complete genome (8,357,362 out of 8,399,976).

**Figure 6:**
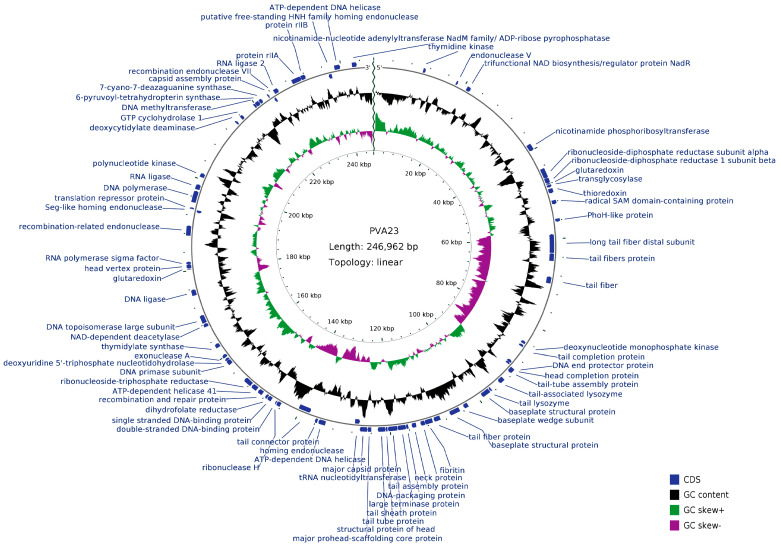
Genome circle map of PVA23. The outermost circle represents ORFs encoded in the genome. The two innermost circles represent GC-skew [(G − C)/(G + C)] and G + C content. The black circle represents the G + C content. The outward direction indicates that the G + C content of this region is larger than the average G + C content of the whole genome, and the inward direction indicates that the G + C content of that region is lower than the average G + C content. The gray circle represents the GC-skew [(G − C)/(G + C)]. The outward direction indicates that the GC-skew [(G − C)/(G + C)] is larger than zero, and the inward direction indicates that the GC-skew [(G − C)/(G + C)] is lower than zero.

**Figure 7:**
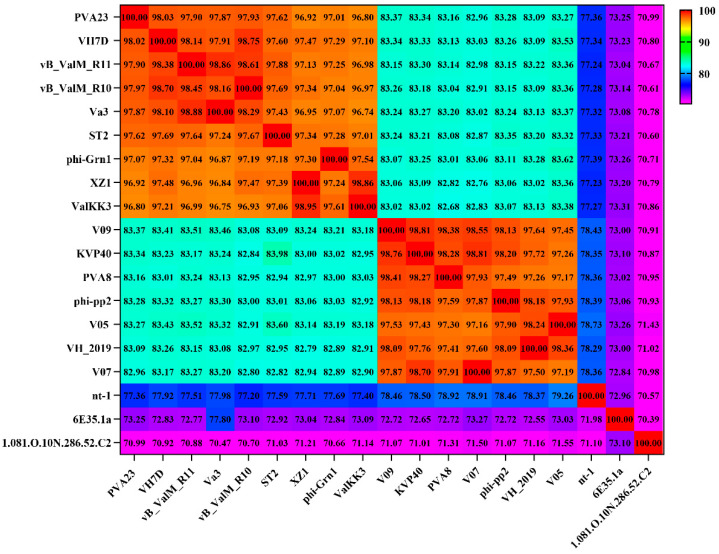
Average nucleotide identity heatmap. The percentage identity values range from 70% (purple) to 100% (red).

The genome circle map of PVA23 was shown in Figure 6. Sequencing analysis showed that the genome was a linear, double-stranded DNA molecule of 246, 962 bp in length with a GC content of 41.25%. The BLASTn comparison result showed that the whole genome sequences of PVA23 had a maximum nucleotide homology with *Vibrio* phage VH7D (98% identity, 97% coverage), and following the higher homology with four other *Vibrio* phages, which were vB_ValM_R10Z, vB_ValM_R11Z, Va3 and phi-ST2 (97% identity, 96% coverage). The ANI heatmap showed that the ANIm percentage identity of PVA23 with VH7D, vB_ValM_R10Z, vB_ValM_R11Z, Va3 and phi-ST2 were 98.02%, 97.97%, 97.90%, 97.87% and 97.62%, respectively (Figure 7). A total of 379 ORFs encoded by the genome were predicted, and 25.33% (96/379) were annotated to have corresponding functions by Blastp analysis against the NCBI nr database. The ORFs that were assigned to the basic phage-related functions were identified, including DNA replication/modification, structure protein, packing protein, metabolism/regulation and host lysis.

In the DNA replication/modification module, several typical enzymes and proteins were found, which were similar to those of other *Vibrio* phages. ORF212 (100% identity to phi-ST2) was a main ORF, encoded single stranded DNA-binding protein. DNA polymerase and DNA helicase were encoded by ORF29 (99.53% identity to phi-ST2) and ORF363 (99.76% identity to VH7D), respectively. ORF251 (100% identity to VH7D) and ORF108 (100% identity to ValKK3) were predicted to encode DNA topoisomerase large subunit and medium subunit, respectively. DNA primase was encoded by ORF227 (100% identity to ValKK3) and ORF264 (100% identity to VH7D) encoded DNA ligase. In addition, phage PVA23 also encoded proteins involved in nucleotide metabolism, including the exonuclease encoded by ORF230 (100% identity to VH7D) and the endonuclease encoded by ORF43 (96.27% identity to ValKK3). These enzymes were responsible for generating deoxyribonucleotides for phage DNA synthesis by hydrolyzing host genomic DNA.

### 3.9. Phylogenetic Analysis and Comparative Genomic Analysis of Phage PVA23

**Figure 8:**
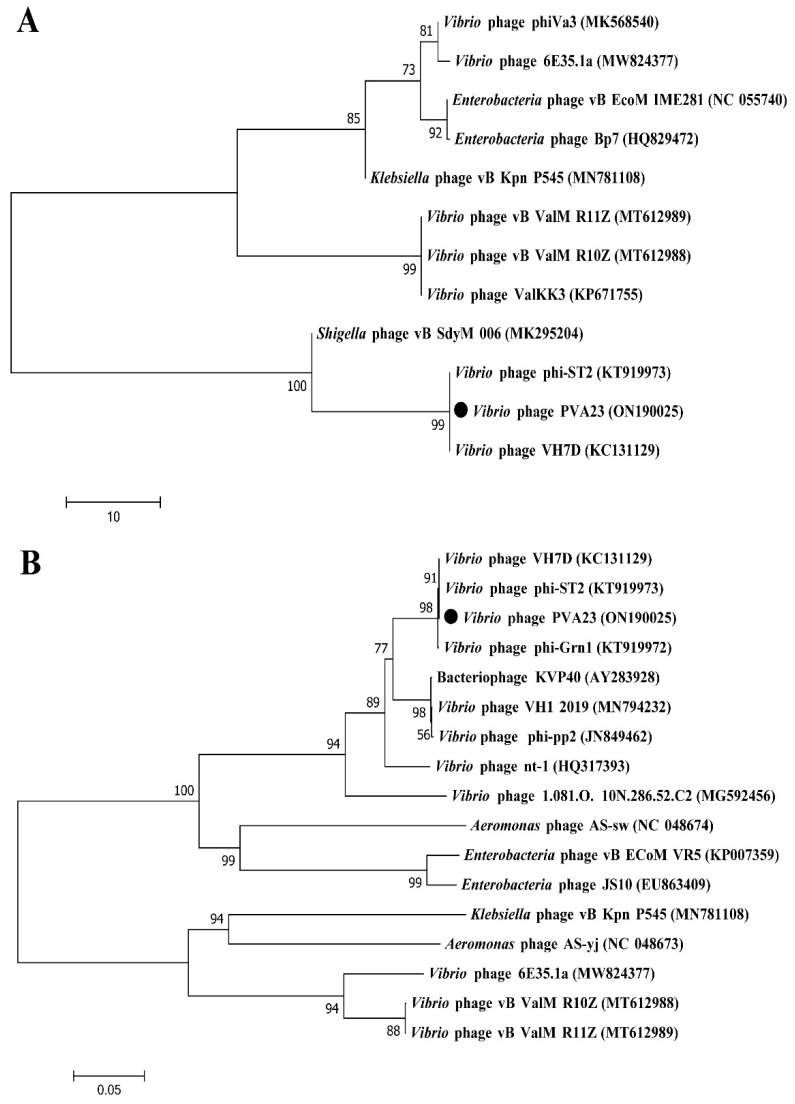
Phylogenetic analysis of phage PVA23. The phylogenetic tree was constructed using the Maximum Likelihood method with 1000 bootstrap replicates. (**A**) Phylogenetic tree was constructed based on the major capsid protein; (**B**) Phylogenetic tree was constructed based on the terminase large subunit.

Structure proteins of phages are commonly used for phages taxonomy; among them, major capsid protein is a highly conserved structural protein [36]. Terminase enzyme can specifically catalyze the packaging reaction of phages, and the larger subunit of terminase holoenzyme is involved in translocation of the cleaved DNA into the empty prohead [36]. Therefore, in order to decipher the evolutionary relationships of PVA23 with the other closely related homologous phages, sequences of the major capsid protein and the large terminase subunit of PVA23 were aligned with that of the other closely related phages downloaded from NCBI database. The phylogenetic trees of the major capsid protein (Figure 8A) and the large terminase subunit (Figure 8B) both confirm that phage PVA23 was more closely related to *Vibrio* phages of phi-ST2 and VH7D, forming a clear clade and showing high homologous coverage (≥91%), while *Vibrio* phages of vB_ValM_R11Z, Va3 and vB_ValM_R10Z were in other clades.

**Figure 9:**
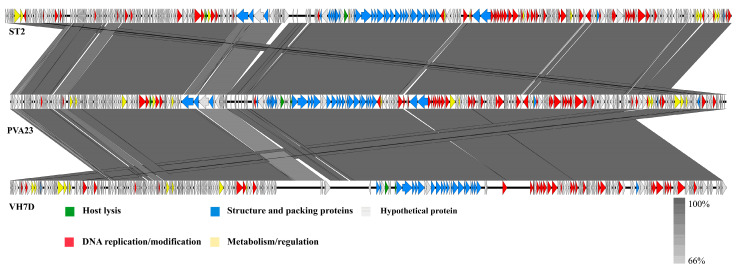
Comparative genome map of a multiple-genome comparison (amino acid level) performed using Easyfig software. Open reading frames (ORFs) are shown as arrows to indicate the direction of transcription and are colored in accordance with their predicted functions: DNA replication/modification (red arrows), structure protein and packing proteins (blue arrows), metabolism/regulation (yellow arrows) and host lysis (green arrows). Gray arrows represent ORFs of unknown functions (hypothetical protein). Gray shading indicates nucleotide identity between sequences (66–100%).

Furthermore, the comparison of whole genomic sequence alignment of PVA23, phi-ST2 and VH7D was conducted, which could enable the depiction of the relationships of the phages at the genomic levels. As aforementioned, the ORFs assigned to basic phage-related functions can be identified and categorized. Therefore, we categorize the genome sequence of PVA23 into five groups: DNA replication/modification, structure and packing proteins, metabolism/regulation, host lysis and hypothetical protein. Similarly, the genome sequences of phi-ST2 and VH7D were downloaded from NCBI and grouped as PVA23. As shown in Figure 9, the genome of PVA23 highly resembled that of phi-ST2 and VH7D, and the nucleotide identity was not less than 66%.

### 3.10. Application Evaluation of Phage PVA23 for Vibrio Control in Laboratory Shrimp Culture Trials

**Figure 10:**
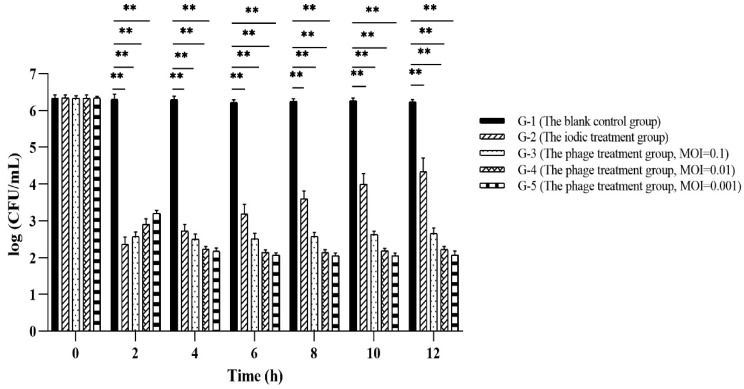
The result of phage PVA23 application in laboratory shrimp culture trials. **, *p* < 0.01.

Results of phage PVA23 application for *Vibrio* control in laboratory shrimp culture trials were shown in Figure 10, and no shrimps died through all the trials. It was observed that in both the phage treatment groups (G-3, G-4 and G-5 in Figure 10) and the iodine disinfectant treatment group (G-2 in Figure 10), the level of *V. alginolyticu* VA15 was reduced by at least three logs at 2 h after supplementation, which is significantly lower than that of the control (G-1 in Figure 10, ~2.14 × 10^6^ CFU/mL). Following this and until 12 h, the level of *V. alginolyticu* VA15 in the iodine treatment group had a tendency to go up, and it eventually bounced up by almost two logs. However, the levels of *V. alginolyticu* VA15 in the three phage treatment groups did not go up, with negligible rebounding observed, which clearly revealed that phage treatment outperformed the iodine treatment in the control of *V. alginolyticu* VA15. The bouncing of VA15 in iodine treatment group may be due to the chemical not being stable with time, e.g., being oxidized when exposed to the continuous airing facilitated by the pump during farming practice. Apparently, the airing process had no effects on the phage, thus no *V. alginolyticu* bouncing was observed with all three treatments receiving phage PVA23. It was concluded that the use of chemical iodine agent could inhibit VA15 in a limited period of time, but its effectiveness was inferior to that of the phage treatments, which could constantly maintain the *V. alginolyticus* VA 15 in a low level during the trials. The above results indicate that PVA23 can rapidly and effectively inhibit the growth of the *V. alginolyticus* VA 15 in shrimp culture practice on laboratory scale, which means that it may have promising application potential in commercial farming practice for the control of the *V. alginolyticus* strains that may cause disease to aquatic animals.

### 3.11. Application Evaluation of Phage PVA23 for Vibrio Control in Shrimp Farming Plants

**Figure 11:**
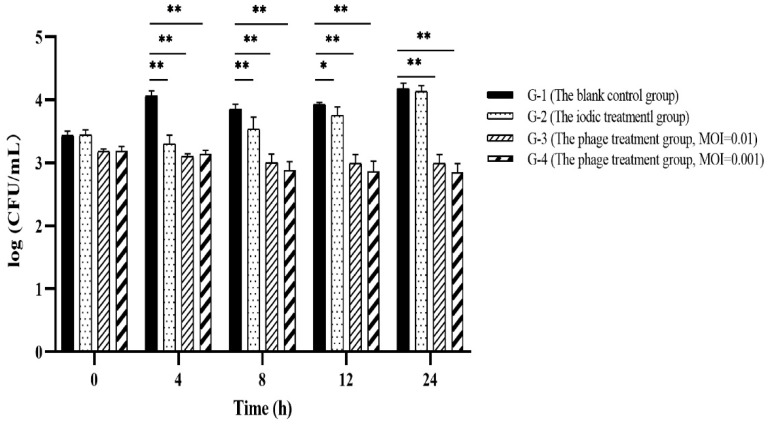
Application evaluation of PVA23 against *Vibrio* in commercial shrimp farming plants. In experiment, it was hard to manage to get identical Vibrio counts in different ponds, the ponds with *Vibrio* ≥ 1000 CFU/mL were selected for the trials and each trial repeated. *Vibrio* counting in the chosen ponds were real, though they might be varied among ponds to some extent. *, *p* < 0.05, **, *p* < 0.01.

To evaluate the effectiveness of PVA23 in the commercial shrimp farming practice, application trials were designed similarly to the lab scale trials, and results were shown in Figure 11. It was found that *Vibrio* content in each blank control (G-1 in Figure 11) were kept constant or increased with time, which reflected the challenge presented by *Vibrio* if no intervention applied. The application of chemical disinfectant iodin (G-2 in Figure 11) showed little effect and the *Vibrio* kept bouncing and elevated to the same level as the control at 24 h. In contrast, *Vibrio* (*V. alginolyticus* for specifical) in the ponds with phages (G-3 and G-4 in Figure 11) maintained a tendency of decreasing during the whole trial, with negligible rebounding observed. 

## 4. Discussion

Since 2006, the FDA approved the direct use of bacteriophages for food applications; thereafter, many commercial products using bacteriophages have been released to control food-borne pathogens [37]. However, the application of phage therapy in aquaculture as well as in clinics is still greatly limited. This may be due to (i) the lack of standardized workflow to prepare phage agents for controlling pathogen infections in practical applications [13], (ii) the lack of documentations supporting the safety, effectiveness and so on 47, (iii) the unpredictable influences on the development of multidrug-resistant bacteria pathogens facilitated by transduction of genetic material through horizontal gene transfer [12,38].

In this work, we demonstrated a systematic workflow for a *V. alginolyticus* lytic bacteriophage, starting from its isolation, identification, then propagation, characterization & sequencing, evaluation, all the way to practical applications. Rather than using *V. alginolyticus* model strains, VA15 and VA17, two *V. alginolyticus* strains isolated from diseased animals in ponds were applied as the hosts for phage isolation, which largely eases the screening of the bacteriophage that kills *Vibrio* isolates and may cause disease in shrimp farming.

With *V. alginolyticus* VA15 as the host strain, a potent *V. alginolyticus* lytic bacteriophage PVA23 was screened and developed in this work. It showed high lytic capability and lytic rate against a panel of *V. alginolyticus* isolates. It also exhibited promising application potentials such as cross-species lysis ability, low MOI, short latent time, large burst size and decent tolerances to adverse conditions. 

Generally, for the phage life cycle, DNA packaging is an important process that requires multiple proteins to interact with the phage genome. Terminase enzymes that can specifically catalyze the packaging reaction of phage are composed of large and small subunits [39]. The large subunit is a multifunctional enzyme, which plays a major role in packaging, and the small subunit blinds specifically to the genome and directs the packaging process. In gene annotating of phage PVA23, the large terminase subunit and the small terminase subunit were found encoded by ORF179 and ORF178, respectively. The annotation results also identified other packaging proteins, such as capsid assembly proteins encoded by ORF337. The phage tail is an important component, and the tail adsorption devices specialize in identifying and binding receptors on the surface of bacteria [36]. In the genome of phage PVA23, the tail-associated ORFs that assigned to tail structure protein were identified and categorized, including tail fiber proteins (ORF125, ORF127, ORF172 and ORF207), tail competition protein (ORF150), tail sheath protein (ORF180), tail tube protein (ORF181), tail-tube assembly protein (ORF157), tail connector protein (ORF206) and baseplate structural proteins (ORF158, ORF165, ORF168 and ORF169), which were involved in tail assembly or in facilitating the penetration of the outer membrane of the host cell upon infection [40]. Except these tail structure proteins, the annotation results also identified other phage structure protein, including the major capsid protein (ORF187), head structural protein (ORF182), neck proteins (ORF174 and ORF175) and head vertex protein (ORF270). There are some ORFs in the genome of PVA23 that were assigned to metabolic/regulated functions. For example, ORF25 and ORF238 were predicted to encode thymidylate kinase and thymidylate synthase, respectively, which were involved in the metabolism of nucleic acids, while several other enzymes predicted were involved in the metabolism of tricarboxylic acid or related compounds. In addition, protein rllA, encoded by ORF349, is one of the major players affecting or avoiding the lysis of another phage, which enables the cell infected by the first invader to avoid or exclude the invasion of another phage virion [41]. For double-stranded DNA bacteriophages, the bacterial lysis is achieved by phage-encoded muralytic enzyme called endolysin that hydrolyze the peptidoglycan (PG) layer present in the bacterial cell wall during the final stage of the phage reproduction events [42]. Based on the specificity, endolysins have at least four different hydrolase activities that degrade the cell wall, which are glycoside transferase, lysozyme, amidase and endopeptidase, respectively [43]. The lytic system of phage PVA23 were composed of tail lysozyme and transglycosylase. ORF160 and ORF164 were predicted to encode tail-associated lysozyme and tail lysozyme, respectively, and transglycosylase was encoded by ORF105. In addition, it was noteworthy that up to thirty-four tRNAs were found in the genome of PVA23 by the tRNAscan-SE, which was on the high end of tRNA numbers found in other similar *Vibrio* phages. The high number of tRNA benefits the synthesis of phage capsid and tail proteins, thus may enhance the phage infection process [44]. In general, virulent phages contain more tRNAs than lysogenic phages. The reason might be that there is a significant association between tRNA distribution and codon usage which leads to an enhanced translational efficiency [45]. The high number of tRNA might also benefit the phage in synthesis of its own proteins [46].

In the genome of phage PVA23, the genes for virulence, antibiotic resistance and lysogeny were not detected, indicating that it could be safely used to control *V. alginolyticus*. The biological and genomic features guarantee that PVA23 is a suitable candidate for phage therapy in the aquaculture farming.

In the evaluation trials, phage PVA23 could rapidly yet effectively reduce the level of *V. alginolyticus* both in the lab and plant shrimp framing applications. The results of phage PVA23 against *Vibrio* (*V. alginolyticus* for specifical) in farming plant trials was consistent with the positive results observed in laboratory tests, which confirmed that PVA23 could serve as an effective biological agent for the control of *V. alginolyticus* in farming plant practice. Not only that, unlike the challenge of shrimp supplemented only with *V. alginolyticus* in the laboratory trial, the bacteria were not added in the field experiments, so any reductions observed were related to *Vibrio* (in general) and both *V. alginolyticus* (specifically). This finding is actually more interesting as it shows that phage PVA23 can also be used to against total *Vibrio* in farming practice, which broadens its application in aquaculture farming. It was noteworthy that phage application evidently outperformed the chemical disinfectant iodine treatment in the prevention of *V. alginolyticus* propagation in pond. Moreover, unlike the immediate rebounds of *V. alginolyticus* in iodine treatment, the levels of *V. alginolyticus* were kept controlled at lower levels than the initial levels, with no rebounding observed. This finding is the first case reporting that phage treatment performs better than the chemical disinfectant in *Vibrio* control in farming practice.

The main challenge of phage therapy observed recently is the resistance issue, which is spontaneous mutation associated and developed by the bacterial defense system [40]. Phage-resistant bacterial variants often emerge when mutations occur spontaneously after treatment with single phage [47,48]. Although phage resistance develops about ten times slower than antibiotic resistance, it does limit the therapeutic applications of phage therapy [12]. 

Phage cocktails, or mixtures of multiple phages, may help overcome the problem by delaying the appearance of resistant variants during phage treatment. It resolves the problems of low efficacy, narrow lysis spectrum and fast emergence of phage-resistant bacterial mutants when using a single phage, making treatment more effective against a specific strain or when targeting multiple strains [13]. However, from the industrial points of view, the phage cocktails might be too costly; as it asks for the preparation of an array of phages, each may need separate production line, not to mention the processing challenges like sterile mixing and the input in formulations.

In this work, fresh cells of *V. alginolyticus* VA15 culture were challenged with PVA23 to check the host resistance development. It was of interest that the addition of phage at higher MOI lead to faster rebounding of the *Vibrio* in pond, though it demonstrated fast killing. This could be that the lytic phage itself functions as the selective pressure, and the higher level of the phage simply leads to the faster presence of stronger resistant bacteria. Therefore, in practice phage application, the optimal MOI of phage should be carefully chosen to ensure achieving faster killing yet slow rebounding of the target *Vibrio*. Low-end MOI was chosen in the evaluation trials in this work, and negligible rebounding in *Vibrio* was noticed. It appeared to be that the choice of phage application at low MOI might be an economic yet practical way to retard the *Vibrio* resistance in aquatic farming. 

In addition, the combination of phages with other antimicrobials derived from probiotics has also been attempted to control the *Vibrio* in the farming practice by our group. Since the antimicrobials killing mechanism are different with that of phage, the application of the combination of phages with other antimicrobials has efficiently reduced the *Vibrio* rebounding and demonstrated decent *Vibrio* control effects (data unpublished).

In conclusion, PVA23, a potent *V. alginolyticus* lytic bacteriophage, was isolated and identified. The phage belongs to the *Myoviridae* family, and it demonstrated a panel of promising application potentials, including high lytic capability, high lytic rate, cross-species lysis ability, low MOI, short latent time, large burst size and decent tolerances to adverse conditions. Genome analysis revealed that there was neither virulence nor antibiotic resistance genes in the genome, thus the phage is environmentally friendly and safe for application. In the pond application trails, PVA23 could rapidly yet effectively reduce the level of *V. alginolyticus* and significantly outperform the chemical disinfectant iodine in *Vibrio* controlling. Furthermore, it was confirmed that the approach for application of phage at low MOI could be a good choice to effectively control the immediate *Vibrio* rebounding caused by resistant *Vibrio* variants or mutants. In this study, a systematic workflow on the discovery and evaluation of a potent phage was exemplified, with PVA23 being successfully evaluated as a promising *Vibrio* control agent in *V. alginolyticus* controlling. Our work confirmed that phage PVA23 can be used as an alternative to antibiotics or chemical disinfectants in shrimp farming practice.

## Figures and Tables

**Table 1:** Primers used in this study for amplification of virulent genes of *V. alginolyticus*.

Gene	Primer	Size (bp)	Reference
*Col*	F: GTACTACGACATTGGCGAAGG R: CCCGACCATACATTTCATACTG	591	[21]
*AspA*	F: GCATGGTACTCACGTAGCGG R: CTTTCACAAGACCAGAAGAGTAACC	146	[21]
*tlh*	F: CGAACGAGAACGCAGACATT R: CTTTGTTGATTTGATCTGGCTG	108	[21]
*tdh*	F: ATAAAGACTATACAATGGCAGCGG R: GAATAGAACCTTCATCTTCACCAAC	138	[21]
*trh*	F: GCCTTTCAACGGTCTTCACAA R: TAACAAACATATGCCCATTTCCG	179	[21]
*FlaA*	F: AATCAATGGAGCGTTTGTCTTC R: GCTACACGTTCTGCTTTTGAGTTAG	253	[21]
*ompW*	F: TCGTGTCACCAAGTGTTTTCG R: CGTGGCTGAATGGTGTTGC	213	[21]
*ompK*	F: ACAGGATCCATGCGTAAATCACTTTT R: ACTCTCGAGTTAGAATTTGTA	800	Gen Bank FJ176400.1
*fur*	F: ATTAACCCTTTGAAGTTCGTGG R: TGACATATACTTTCCCGTTGGATC	111	[21]
*gyrB*	F: ATTGAGAACCCGACAGAAGCGAAG R: CCTAATGCGGTGATCAGTGTTACT	340	[21]
*toxR*	F: GGATTCAACCAAATCTCCAGAGT R: GCTCAATAGAAGGCAACCAGTT	434	[21]
*toxS*	F: GCCGTATCTATCCTTTTCAGTGG R: GCCTTGTGCGAACAGTTTG	228	[21]

**Table 2:** Host range of the isolated phages to the *V. alginolyticus* strains.

Strains	Isolation Source	PVA21	PVA22	PVA23	PVA24	PVA25	PVA26
17749	ATCC	+	−	−	−	+	−
VA0	Ningbo, Zhejiang	−	−	++	−	+++	−
VA1	Ningbo, Zhejiang	++	−	+++	−	+	+++
VA3	Nantong, Jiangsu	−	−	+	+++	−	−
VA5	Rudong, Jiangsu	−	+++	+	++	−	−
VA6	Rudong, Jiangsu	−	−	+++	+	−	−
VA7	Rudong, Jiangsu	+++	−	+++	+++	−	−
VA8	Ningbo, Zhejiang	−	++	−	−	++	−
VA9	Ningbo, Zhejiang	−	−	+++	−	−	
VA10	Ningbo, Zhejiang	++	−	+++	++	−	
VA11	Ningbo, Zhejiang	−	−	+++	−	−	
VA12	Ningbo, Zhejiang	−	++	−	−	+++	
VA13	Nantong, Jiangsu	+		+	−	−	++
VA14	Nantong, Jiangsu	−		+++	−	−	
VA15	Nantong, Jiangsu	−	+++	+++	+++	+++	−
VA16	Ningbo, Zhejiang	−	−	+	++	−	
VA17	Ningbo, Zhejiang	+++	−	++	++	+++	+++
VA18	Ningbo, Zhejiang	−	−	++	+	++	
VA19	Nantong, Jiangsu	+++	−	−	−	++	
VA20	Nantong, Jiangsu	−	−	−	++	−	
VA21	Nantong, Jiangsu	−	++	+		++	
VA22	Nantong, Jiangsu	−	−	++		−	++
VA23	Qingdao, Shandong	++	−	−	+++	++	
VA24	Qingdao, Shandong	−	−	++		+	
VA25	Qingdao, Shandong	−	++	+++		−	
VA26	Qingdao, Shandong	+++	−	−	++	−	
VA27	Qingdao, Shandong	−	−	+++		−	
VA28	Qingdao, Shandong	−	+	−	++	−	
VA29	Qingdao, Shandong	−	−	++		++	
VA30	Qingdao, Shandong	+	−	+		−	+
VA31	Rizhao, Shandong	−	+++	−	+	−	
VA32	Rizhao, Shandong	−	−	−	+++	+	
VA33	Rizhao, Shandong	−	−	−		++	
VA34	Rizhao, Shandong	−	−	++		−	
VA35	Rizhao, Shandong	−	++	−	++	−	
VA36	Rizhao, Shandong	−	−	++		−	
VA37	Rizhao, Shandong	++	−	−	+	−	
VA38	Nanning, Guangxi		−	−	+	++	
VA39	Nanning, Guangxi	++	−	−	−	+++	
VA40	Nanning, Guangxi		−	++	−	−	+++
VA41	Nanning, Guangxi			+	+++		

+++, clear plaques; ++, plaques with slightly turbidity; +, plaques with heavy turbidity; −, no plaques formed. The growth conditions were all 2216E agar and broth at 37 °C.

**Table 3:** Host range of the isolated phages to the *V. alginolyticus* strains.

Strains	Isolation Source	Lysis	EOP
17749	ATCC	−	−
VA0	Ningbo, Zhejiang	++	0.345 ± 0.03
VA1	Ningbo, Zhejiang	+++	0.887 ± 0.04
VA3	Nantong, Jiangsu	+	−
VA5	Rudong, Jiangsu	+	0.012 ± 0.02
VA6	Rudong, Jiangsu	+++	0.906 ± 0.04
VA7	Rudong, Jiangsu	+++	0.912 ± 0.05
VA8	Ningbo, Zhejiang	−	−
VA9	Ningbo, Zhejiang	+++	1.078 ± 0.07
VA10	Ningbo, Zhejiang	+++	0.975 ± 0.06
VA11	Ningbo, Zhejiang	+++	1.033 ± 0.09
VA12	Ningbo, Zhejiang	−	−
VA13	Nantong, Jiangsu	+	−
VA14	Nantong, Jiangsu	+++	0.865 ± 0.03
VA15	Nantong, Jiangsu	+++	1
VA16	Ningbo, Zhejiang	+	0.112 ± 0.01
VA17	Ningbo, Zhejiang	++	0.294 ± 0.02
VA18	Ningbo, Zhejiang	+	0.178 ± 0.01
VA19	Nantong, Jiangsu	−	−
VA20	Nantong, Jiangsu	−	−
VA21	Nantong, Jiangsu	+	0.049 ± 0.01
VA22	Nantong, Jiangsu	++	0.543 ± 0.03
VA23	Qingdao, Shandong	−	−
VA24	Qingdao, Shandong	++	0.613 ± 0.02
VA25	Qingdao, Shandong	+++	0.847 ± 0.05
VA26	Qingdao, Shandong	−	−
VA27	Qingdao, Shandong	+++	0.931 ± 0.04
VA28	Qingdao, Shandong	−	−
VA29	Qingdao, Shandong	++	0.710 ± 0.03
VA30	Qingdao, Shandong	+	0.211 ± 0.02
VA31	Rizhao, Shandong	−	−
VA32	Rizhao, Shandong	−	−
VA33	Rizhao, Shandong	−	−
VA34	Rizhao, Shandong	++	0.435 ±0.03
VA35	Rizhao, Shandong	−	−
VA36	Rizhao, Shandong	++	0.236 ± 0.04
VA37	Rizhao, Shandong	−	−
VA38	Nanning, Guangxi	−	−
VA39	Nanning, Guangxi	−	−
VA40	Nanning, Guangxi	+	0.077 ± 0.01
VA41	Nanning, Guangxi	+	−

+++, clear plaques; ++, plaques with slightly turbidity; +, plaques with heavy turbidity; −, no plaques formed. The growth conditions were all 2216E agar and broth at 37 °C.

**Table 4:** Host ranges of phage PVA23 to other *Vibrio* strains.

Bacterial Species	Medium	Total Number of Strains	Lysis Number of Strains
*Vibrio parahaemolyticus*	2216E	28	2
*Vibrio*. spp.	2216E	42	3
*Bacillus subtilis*	LB	2	0
*Bacillus licheniformis*	LB	2	0
*Lactic acid bacteria*	MRS	2	0
*Photosynthetic bacteria*	*Photosynthetic bacteria* medium	2	0
*Saccharomyces bacteria*	PDA	2	0

**Table 5:** The result of the BIMF of *V. alginolyticus* VA15 to phage PVA23.

	Assumed Insensitive Colonies in Plates	Determined Insensitive Colonies by Standard Spot Test	Total Colonies without Phage	The BIMF
Phage group	10 ± 1	9 ± 1		(8.48 ± 0.63) × 10^−7^
Control group			(1.06 ± 0.04) × 10^7^	

## Data Availability

The datasets generated for this study can be found in the phage was preserved in the China General Microbiological Culture Collection Center (Beijing, China) with CGMCC number of No.24119. The complete genome sequences of the phage PVA23 have been submitted to the NCBI GenBank database with accession number of ON190025.
